# CircRNA_000864 Upregulates B-cell Translocation Gene 2 Expression and Represses Migration and Invasion in Pancreatic Cancer Cells by Binding to miR-361-3p

**DOI:** 10.3389/fonc.2020.547942

**Published:** 2020-12-23

**Authors:** Linsheng Huang, Junxiang Han, Huifan Yu, Jialing Liu, Lili Gui, Zhengkun Wu, Xinxu Zhao, Shiqi Su, Gaohang Fu, Fei Li

**Affiliations:** ^1^Department of Hepatopancreatobiliary Surgery, Taihe Hospital, Hubei University of Medicine, Shiyan, China; ^2^Hubei Key Laboratory of Wudang Local Chinese Medicine Research, Hubei University of Medicine, Shiyan, China

**Keywords:** pancreatic cancer, circRNA_000864, microRNA-361-3p, Btg2, proliferation, migration, invasion, apoptosis

## Abstract

**Background:**

Pancreatic cancer is a fatal disease with a very poor prognosis due to its characteristic insidious symptoms, early metastasis, and chemoresistance. Circular RNAs (circRNAs) are involved in the development of pancreatic cancer.

**Aim:**

Hence, the aim of this study is to elucidate the mechanism of circRNA_000864 in regulating BTG2 expression in pancreatic cancer by binding to miR-361-3p.

**Methods:**

CircRNA_000864, miR-361-3p, and BTG2 expression patterns in the pancreatic cancer tissues were detected by RT-qPCR. Correlation among circRNA_000864, miR-361-3p, and BTG2 was evaluated by RNA-pull down assay, RNA Immunoprecipitation assay, and dual-luciferase reporter gene assay. After ectopic expression and depletion experiments, 5-ethynyl-2′-deoxyuridine assay, Transwell assay, and flow cytometry were employed to assess the cell proliferation, migration and invasion, cell cycle, and apoptosis. Xenotransplantation of nude mice was conducted to detect the effects of circRNA_000864, miR-361-3p, and BTG2 on tumor growth.

**Results:**

CircRNA_000864 and BTG2 were poorly expressed, and miR-361-3p was highly expressed in the pancreatic cancer tissues. CircRNA_000864 bound to miR-361-3p could target BTG2. Cell proliferation, migration, and invasion were inhibited, and the cell cycle arrest and apoptosis were stimulated after overexpression of circRNA_000864 or BTG2 or downregulation of miR-361-3p. Overexpression of circRNA_000864 or downregulation of miR-361-3p also decreased the tumor growth *in vivo*.

**Conclusions:**

Conjointly, our findings elicited that the overexpression of circRNA_000864 could promote BTG2 expression to inhibit pancreatic cancer development by binding to miR-361-3p, which represents an appealing therapeutic target for the treatment of pancreatic cancer.

## Introduction

Pancreatic cancer, as the seventh leading cause of cancer associated with mortality in both men and women, has received an increasing attention worldwide (1). Research has observed an association between familial genetics and the morbidity of pancreatic cancer (2). Moreover, chronic pancreatitis, which is prevalent in patients with pancreatic cancer, is concurrent with orodigestive cancer-related fatalities (3). Conventional therapeutic protocols for pancreatic cancer include chemotherapy and radiotherapy; however, some novel therapeutic methods such as genomics, immunotherapies, and strategies to target tumor stroma are gaining significance in treating pancreatic cancer (4, 5).

Circular RNAs (circRNAs), a newly discovered line of non-coding RNAs, have been highlighted to function as gene regulators and with highly differentiated roles in various human diseases (6). Existing research has elicited a correlation between circRNAs and pancreatic cancer. Silencing circ_0030235 by siRNAs principally suppresses the cell proliferation, migration, and invasion of pancreatic cancer (7). Moreover, circ-LDLRAD3 was highly expressed in pancreatic cancer, while silencing circ-LDLRAD3 suppressed the progression of pancreatic cancer (8). In addition, circRNA could regulate the gene expression by serving as a microRNA (miRNA) sponge in pancreatic cancer (9). In our study, the role and significance of circRNA_000864 in pancreatic cancer was investigated. It has been shown in the circbase^1^ that circRNA_000864 (circRNA ID as hsa_circ_0000662) is located at the position of chr16:398402–398484 with a genomic length of 82. Furthermore, the expression dataset analysis revealed that circRNA_000864 was poorly expressed in pancreatic cancer and was bound to miR-361-3p. Similarly, circRNA 100146 exercises its oncogenic effect on non-small cell lung cancer (NSCLC) by interacting with miR-361-3p (10). An existing study elucidated that miR-361-3p downregulation radically inhibits the cell migration and invasion of pancreatic ductal adenocarcinoma (PDAC) cells (11). An existing study elicited that B-cell translocation gene 2 (BTG2) was targeted by miR-361-3p (12). As an antiproliferation gene, BTG2 downregulation is indicated in malignant cell behavior and poor treatment outcome in solid tumors, which indicates the functionality of BTG2 as a prognostic biomarker in multiple tumors (13). Overexpression of BTG2 induces triple-negative breast cancer (TNBC) cell apoptosis by suppressing miR-25-3p (14). Based on the aforementioned literature, we hypothesized the significance of novel circRNA circRNA_000864 in pancreatic cancer *via* BTG2 and miR-361-3p. The current study aims to determine the interaction between circRNA_000864, miR-361-3p, and BTG2 in pancreatic cancer, and their effects on pancreatic cancer cell proliferation, invasion, and apoptosis.

## Materials and Methods

### Ethics Statement

All experimentation protocols were conducted in strict accordance with the *Declaration of Helsinki*. The study protocol was conducted with the approval of the Ethics Committee of Hubei University of Medicine. All patients provided written informed consent prior to enrollment.

Animal use and experimental procedures were conducted with the approval of the Experimental Animal Ethics Committee of Hubei University of Medicine. All experimental animal operating procedures are in compliance with the guidelines issued by the United States National Institutes of Health laboratory (NIH) for animal care and usage.

### Bioinformatics Prediction

Pancreatic cancer-related microarray data were retrieved from the Gene Expression Omnibus (GEO) database,^2^ which comprised of the circRNA dataset GSE79634 and gene expression dataset GSE16515 for analysis of the differential expressions. The “limma” package of R language programming was employed for standardization of the microarray data and screening of the differentially expressed molecules. “Pheatmap” package^3^ was adopted to construct the expression heatmap for the differentially expressed circRNA. After screening of the differentially expressed circRNA, the miRNAs that could potentially target circRNA were predicted in the CircInteractome database.^4^ TargetScan,^5^ RNA22,^6^ miRWalk,^7^ miRSearch,^8^ starBase,^9^ and HOCTAR^10^ were employed to predict the target genes of miRNA. The “UpSetR” package of R language programming^11^ was adopted to compare the differences between the prediction results of target genes and the differential gene results in GSE16515.

### Sample Selection

From a period spanning from 2010 to 2015, 59 pancreatic cancer specimens were resected after pathological verification by the Department of Hepatopancreatobiliary Surgery of Taihe Hospital, Hubei University of Medicine. Pancreatic cancer and the adjacent normal tissues (more than 5 cm away from the cancerous site) were also harvested. The patients consisted of 36 males and 23 females, with the mean age of 58.59 ± 62.0 years (ranging 44–70 years), among which six cases were classified as stage I, nine cases as stage II, 33 cases as stage III, and 11 cases as stage IV. No patient had a history of other malignancies, severe infections, cognitive impairment, poor compliance, or inability to comprehend the research process. The collected sample tissues were divided into two portions. One portion was preserved in the liquid nitrogen tank immediately for RNA and protein extraction. The other portion was fixed with paraformaldehyde and embedded in paraffin for subsequent experimentation.

### Cell Culture

Pancreatic cancer cell lines AsPC-1, MiaPaCa-2, PANC-1, and HPAC were cultured in Dulbecco’s modified Eagle medium (DMEM; Invitrogen, Carlsbad, CA, United States) containing 10% fetal bovine serum (FBS) and 100 U/ml of penicillin. The normal pancreatic epithelial cell line HPDE6C7 was incubated using DMEM (Invitrogen) containing 5 ng/ml of epidermal growth factors and 50 μg/ml of bovine pituitary extracts. All cells were cultured in a 5% CO_2_ incubator at 37°C. After attaining 90% confluence, the cells were trypsinized and passaged, and RT-qPCR was employed for the final screening. The aforementioned cell lines were purchased from Shanghai Biological Technology Co., Ltd. enzyme research (Shanghai, China),^12^ and the cell line contamination verification was conducted to show no contamination.^13^

### Cell Transfection

The screened pancreatic cancer cell lines were collected and transfected with overexpression (oe)-circRNA_000864-1 plasmid, oe-circRNA_000864-2 plasmid, miR-361-3p mimic, miR-361-3p inhibitor, short hairpin (sh)-BTG2 plasmid, and their control oe-NC plasmid, mimic-NC, inhibitor-NC, and sh-NC plasmid. oe-circRNA_000864, miR-361-3p mimic, miR-361-3p inhibitor, and sh-BTG2 were purchased from Guangzhou RiboBio Co., Ltd. (Guangdong, China) (sh-NC: 5′-GCTACACAAATCAGCGATTT-3′; sh-BTG2: 5′-GGACGCACTGACCGATCATTA-3′). The cells were seeded into a 24-well plate to facilitate the cell confluence to 50–60% after transfection. The pancreatic cancer cells were transfected according to the provided instructions of Lipofectamine^TM^ (Invitrogen). After culture in a 5% CO_2_ incubator at 37°C for 6–8 h, the medium was replaced by complete medium.

### Fluorescence *in situ* Hybridization

Fluorescence *in situ* hybridization (FISH) was performed by applying the circRNA_000864 sequence and specific probes of miR-361-3p. The cy5-labeled probe was specific for circRNA_000864, and the farm-labeled probe was specific for miRNA. The nuclei were stained in 4′,6-diamidino-2-phenylindole (DAPI). All the procedures were carried out in accordance with the manufacturer’s operating manual (Shanghai GenePharma Co., Ltd., Shanghai, China). All the images were acquired on a Zeiss LSM880 NLO (2 + 1 with BIG) confocal microscope system (Leica Microsystems, Mannheim, Germany).

### RT-qPCR and RNase R Treatment

After 24 h of transfection, the Trizol kit (15596026, Invitrogen) was adopted to extract the total RNA content. The extracted RNA content was then reverse transcribed into cDNA according to the provided instructions of the PrimeScript RT reagent kit (TaKaRa, Code NO. RR047A, Shiga, Otsu, Japan). Primers for circRNA_000864, miR-361-3p, BTG2, U6, and GAPDH were designed and synthesized by Shanghai Sangon Biotechnology Co. Ltd. (Shanghai, China) (Table 1). RT-qPCR was performed using the 7500-type fluorescence quantitative PCR instrument (ABI Company, Oyster Bay, NY, United States) in strict accordance with the provided instructions of EasyScript First-Strand cDNA Synthesis SuperMix (AE301-02, TransGen Biotech, Beijing, China). The 2^–ΔΔCt^ value represented the gene expression ratio between the experimental group and the control group (15).

**TABLE 1:** Primer sequences of RT-qPCR.

**Gene**	**Forward primer (5′–3′)**	**Reverse primer (5′–3′)**
circRNA_000864	TAGACAAGGGACAGC AGTGTG	CATTGCACACACTTT ACCAGCC
miR-361-3p	CTGCACTCCCCCACCTG	GTGCAGGGTCCGAGGT
BTG2	AGGGCCGTCTTTC TTCTACTC	CTTCCGAGAGCGT AGAGGGG
GADPH	CTGGGCTACAC TGAGCACC	AGTGGTCGTTGA GGGCAATG
U6	TGCGGGTGCTCG CTTCGGCAGC	CCAGTGCAGGGT CCGAGGT

*CircRNA, circular RNA; BTG2, B-cell translocation gene 2; GADPH, glyceraldehyde-3-phosphate dehydrogenase; R, reverse; F, forward.*

### Western Blot Analysis

Cells were lysed by cell lysis buffer (P0013, Beyotime Biotechnology, Shanghai, China), collected into a 1.5-ml EP tube, centrifuged at 12,000 rpm at 4°C for 15 min, followed by supernatant collection. The total protein concentration was determined using the BCA detection kit (P0012-1, Beyotime Biotechnology). Afterward, the proteins were added with loading buffer, boiled for 5 min, and then transferred to a PVDF membrane (Millipore, Billerica, MA, United States) through 10% SDS-PAGE gel. The membrane was sealed with 5% skimmed milk powder at room temperature for 1 h and incubated overnight with TBST diluted primary antibodies to BTG2 (ab197362; 1:1,000, Abcam) and β-actin (ab179467, 1:5,000, Abcam) at 4°C. The membrane was further incubated with the diluted horseradish (HRP)-labeled secondary goat anti-rabbit antibody (ab205718; 1:10,000, Abcam) for 1 h. Image was developed using ECL under X-ray exposure and photographed. Absorbance analysis of the bands was performed using a gel imaging analysis system. The experiment was conducted three times independently.

### RNA-Pull Down Assay

Pancreatic cancer cell AsPC-1 was transfected with Wt-bio-miR-361-3p and Mut-bio-miR-361-3p, which were labeled using 50 nM biotin. After 48 h, the cells were collected and rinsed using phosphate buffer saline (PBS). Then, the cells were incubated in a specific lysis (Ambion, Austin, Texas, United States) for 10 min and centrifuged at 14,000 × *g*, after which the supernatant was collected. The protein lysate was incubated with the M-280 streptavidin beads (S3762, Sigma-Aldrich Chemical Company, St Louis, MO, United States), which were precoated with RNase-free bovine serum albumin (BSA) and yeast tRNA (TRNABAK-RO, Sigma-Aldrich Chemical Company). The beads were incubated for 3 h at 4°C, rinsed twice with the precooled lysis, three times with low salt buffer, and once with the high salt buffer. The bound RNA content was purified by means of Trizol, after which the circRNA_000864 expression was detected by RT-qPCR.

### RNA Immunoprecipitation Assay

Pancreatic cancer cells were lysed with the lysis buffer [25 mM Tris–HCl (pH 7.4), 150 mM NaCl, 0.5% NP-40, 2 mM ethylenediamine tetraacetic acid (EDTA), 1 mM NaF, and 0.5 mM dithiothreitol] containing RNasin (Takara Bio Inc., Otsu, Shiga, Japan) and protease inhibitors (B14001a, Roche, United States). Next, the cells were centrifuged at 12,000 × *g* for 30 min, and the supernatant was collected. Then anti-human Argonaute 2 (Ago-2) magnetic beads (BMFA-1, Biomarker, Beijing, China) were added, and the anti-IgG magnetic beads were supplemented as control. After a regimen of incubation at 4°C for 4 h, the beads were rinsed using the wash buffer solution [50 mM Tris-HCl, 300 mM NaCl (pH 7.4), 1 mM MgCl_2_, and 0.1% NP-40] three times. The RNA content was extracted from the magnetic beads using Trizol, and circRNA_000864 and miR-361-3p expression was detected by means of RT-qPCR.

### 5-Ethynyl-2′-Deoxyuridine Staining

Cell proliferation experiments were performed using the 5-ethynyl-2′-deoxyuridine (EdU) assay kit (C10310, Guangzhou RiboBio Co., Ltd., Guangdong, China). Cells in the logarithmic growth phrase were seeded in a 96-well plate at a density of 1 × 10^4^ cells/well. When the cells adhered to the wall and progressed into the normal growth phase, 100 μl of EdU medium (50 μM) was added to each well for a regimen of 2-h incubation at 37°C. After fixation with 40 g/l of paraformaldehyde for 20 min, the cells were incubated with 2 mg/ml of glycine for 10 min and rinsed twice with PBS. Each well was supplemented with 100 μl of penetrant (PBS with 0.5% Triton X-100; T8200, Beijing Solarbio Science & Technology Co., Ltd., Beijing, China) and incubated for 10 min. Then the cells of each well were supplemented with 100 μl of the Apollo staining reaction solution and incubated in a greenhouse for 30 min in dark conditions. The cells were cultured with the Hoechst33342 reaction solution at room temperature for 30 min, and then rinsed twice with 0.5% Triton X. The observations were photographed under an inverted fluorescence microscope, and the number of EdU-labeled cells was recorded. Cells with the nucleus stained red were labeled as positive cells, and the number of positive and negative cells in the randomly selected three fields was counted under a microscope. EdU labeling rate (%) = number of positive cells/(number of positive cells + number of negative cells) × 100%.

### Transwell Assay

Cells were supplemented with 200 μl of serum-free medium in the apical chamber (3 × 10^4^ cells/well). Then 500 μl of fresh medium containing 10% FBS was added in the basolateral chamber. The cells were then incubated at 37°C with 5% CO_2_ for 24 h for migration assay. For the invasion assay, the procedure was the same as above with an exception that the Transwell apical chamber was precoated with 200 mg/ml of Matrigel. Following 48 h of incubation, the migration and invading cells of the basolateral chamber were stained with 0.1% crystal violet. Images of the pancreatic cancer cells were documented under a phase contrast microscope.

### Flow Cytometry

Annexin V-fluorescein isothiocyanate (FITC)/propidium iodide (PI) double staining kit (556547, Shanghai Solja Technology Co., Ltd., Shanghai, China) was employed to detect the apoptosis rate of the pancreatic cancer cells after 48 h of transfection. Cells were centrifuged at 2,000 rpm for 5 min. The collected cells were then resuspended in 1 × PBS, and centrifuged at 200 rpm for 5–10 min. Then 300 μl of 1 × binding buffer was added to suspend the cells, and 5 μl of Annexin V-FITC was added and mixed, and the mixture was incubated for 15 min at room temperature in dark conditions. Then 5 μl of PI was added for incubation in dark conditions for 5 min. After cells were placed on a flow cytometer, FITC was detected at the excitation wavelengths of 480 and 530 nm, and PI was detected above 575 nm.

The transfected cells were trypsinized to prepare a single-cell suspension, and centrifuged, followed by removal of the supernatant. Precooled 70% ethanol was added to fix the cells overnight at 4°C, after which the cells were resuspended. After centrifugation, the cells were resuspended in 100 μl of PBS. RNase was added to adjust the final concentration to 1 mg/ml, and the cells were submersed in 37°C water for 30 min. The PI staining solution was added to adjust the final concentration to 50 μg/ml, and the cells were stained at 4°C for 40 min in dark conditions. The DNA content of the cell cycle was measured when the laser wavelength was above the excitation wavelength of 575 nm, and the percentage of the cell cycle was calculated.

### Dual-Luciferase Reporter Gene Assay

A binding site analysis between circRNA_000864 and miR-361-3p as well as between miR-361-3p and BTG2 was performed using the biological prediction website, and a fragment sequence containing the action sites was obtained. The full length of circRNA_000864 (forward primer: 5′-AACGGCGC CCGCTCCACACTGCTGTCCCTTGTCTAT-3′; reverse primer: 5′-AGTTGGCGGGCGCCTGTTGGCTGGTAAAGTGTGTGCA ATGT-3′) and the 3′ untranslated region (UTR) region of BTG2 (forward primer: 5′-AACGGCGCCCGCGCCCTTCC GCCCCCGCCCTGGGCGC-3′; reverse primer: 5′-AGTTG GCGGGCGCACATTTGTCCATAAGCTGTACATTC-3′) were separately cloned into the pmirGLO (E1330, Promega Corporation, Madison, WI, USA) luciferase vector and named as pcircRNA_000864-wild type (Wt) and pBTG2-Wt. The pcircRNA_000864-mutant (MUT) and pBTG2-Mut vectors were, respectively, constructed; the internal reference was pRL-TK vector (E2241, Promega Corporation, Madison, WI, United States), which expressed the Renilla luciferase. Luciferase vectors (CRL-1415, ATCC, Manassas, VA, United States) were cotransfected with the mimic NC and miR-361-3p mimic into the pancreatic cancer cell AsPC-1. The Dual Luciferase Reporter Gene Assay Kit (GM-040502A, Shanghai qcbio Science&Technologies Co., Ltd., Shanghai, China) was applied to measure the fluorescence intensity at 560 nm (firefly RLU) and 465 nm (sea kidney RLU), and the firefly RLU/sea kidney RLU ratio was determined according to the binding strength.

### Immunohistochemistry

The 10% formaldehyde fixed specimens were embedded in paraffin and sliced into 4-μm-thick sections. Tissue sections were subjected to incubation in a 60°C incubator for 1 h, dewaxed by conventional xylene, dehydrated using gradient alcohol, and incubated in 3% H_2_O_2_ (Sigma-Aldrich Chemical Company) for 30 min at 37°C. After a rinse with PBS, the tissue sections were boiled in 0.01 M citrate buffer at 95°C for 20 min and rinsed with PBS after waning to room temperature. Next, a blockade was conducted using the normal sheep serum working solution at 37°C for 10 min. Primary anti-rabbit anti-BTG2 (ab85051, 1:100, Abcam, Cambridge, United Kingdom) was added to the cells and reacted at 4°C for 12 h. After a rinse with PBS, the corresponding biotin-labeled secondary goat anti-rabbit antibody (ab150077, 1:100, Abcam) was added drop-wise and facilitated to react at room temperature for 10 min. After a thorough rinse, the peroxidase HRP-labeled streptavidin (S-A) working solution was added and facilitated to react at room temperature for 10 min. Diaminobenzidine (DAB) served as a color-developing reagent, stored at room temperature in dark conditions for 8 min, and then rinsed under tap water. The tissue sections were stained with hematoxylin, dehydrated, transparent, sealed, and observed under a light microscope. The number of positive cells was counted using the Japanese Nikon image analysis software, and three equal-area non-repetitive fields (200 times) were selected for each slice to calculate the number. The criteria for immunohistochemical staining were as follows: ABCF2 (stained area above 25% was positive cells) with significant brown or brownish yellow particles appearing in the cytoplasm. Positive expression rate = number of positive cases/total number of cases (16).

### Xenotransplantation of Nude Mice

Approximately 1 × 10^7^ cells were injected subcutaneously into the armpits of BALB/C nude mice (half male and half female, 78 in total, 5–6 weeks old, 12.40–19.79 g). Tumor growth was monitored weekly by measuring the width (W) and length (L) with a caliper, and the volume (V) of the tumor was calculated based on the formula V = (W2 × L)/2. Four weeks after the injection, the mice were euthanized, and the tumor weight was measured.

### Statistical Analysis

SPSS 21.0 (IBM Corp., Armonk, NY, United States) software was adopted for statistical analysis. Measurement data were expressed as mean ± standard deviation, and the normal distribution and homogeneity of variance were first tested. For data conforming to normal distribution and homogeneity of variance, the paired *t*-test was employed to compare data within a group, while the unpaired *t*-test was used for comparisons between two groups. One-way analysis of variance (ANOVA) (Dunnett’s *post hoc* test or Tukey’s *post hoc* test) was adopted for comparison among multiple groups. Repeated measures ANOVA (Tukey’s *post hoc* test) was used to compare the data at different time points. The correlation between two groups was analyzed using the Pearson correlation. In all statistical data, a value of *p* < 0.05 was indicative of a statistically significant difference.

## Results

### CircRNA_000864/miR-361-3p/BTG2 Axis May Participate in Pancreatic Cancer Development

**FIGURE 1:**
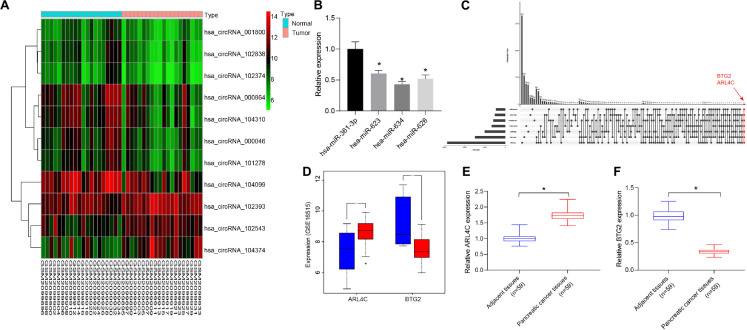
CircRNA_000864 may affect pancreatic cancer development by mediating B-cell translocation gene 2 (BTG2) through miR-361-3p. **(A)** Heatmap of 10 differentially expressed circular RNAs (circRNAs) in pancreatic cancer circRNA gene expression dataset GSE79634. The abscissa indicates the sample number, the ordinate indicates the differentially expressed gene, and the right upper histogram is the color gradation. Each rectangle in the figure corresponds to one sample expression value. **(B)** Expression of miRNAs in pancreatic cancer tissues (*n* = 59). **(C)** The comparison between the target gene predicting results of miR-361-3p in TargetScan, RNA22, miRWalk, miRSearch, starBase, and HOCTAR, and the results of differential analysis of GSE16515. The red dot-line indicates that there are two overlapped genes. **(D)** ARL4C and BTG2 expression in the GSE16515 gene expression datasets. Red indicates tumor tissue, and blue indicates normal tissue. **(E)** ARL4C BTG2 expression in pancreatic cancer tissues. **(F)** BTG2 expression in pancreatic cancer tissues. *p < 0.05 *vs.* adjacent normal tissues. The above data were all measurement data and expressed as mean ± standard deviation. The paired *t*-test was employed to compare data between two groups. One-way analysis of variance (ANOVA) was adopted for comparison among multiple groups.

Differential analysis of the pancreatic cancer circRNA gene expression dataset GSE79634 was performed. Heatmap of the first 10 differentially expressed circRNAs was drawn (Figure 1A), with a lower circRNA_000864 expression in pancreatic cancer tumor tissues than that in adjacent normal tissues. circRNA_000864 presented with the lowest p value, suggesting its most significant differential expression, and was thus selected for subsequent experiments.

The CircInteractome database predicted four miRNAs as potential targets of circRNA_000864, which were as follows: hsa-miR-361-3p, hsa-miR-634, hsa-miR-623, and hsa-miR-626, among which the expression of miR-361-3p was the highest in pancreatic cancer (Figure 1B). An existing study reported that the overexpression of miR-361-3p stimulated the migration and invasion of pancreas cancer cells *in vitro* (11). Thus, it was speculated that circRNA_000864 was most likely to affect pancreatic cancer by targeting miR-361-3p.

In addition, the target genes of miR-361-3p were predicted using TargetScan, RNA22, miRWalk, miRSearch, starBase, and HOCTAR databases, and then the results were compared with that of the differential analysis of GSE16515. The two overlapped genes were obtained: ARL4C and BTG2 (Figure 1C). As shown in Figure 1D, BTG2 was poorly expressed in pancreatic cancer, while ARL4C was highly expressed. Since miR-361 was highly expressed in pancreatic cancer, and miRNAs usually inhibit the expression of target genes, we speculated that miR-361-3p may target BTG2 in pancreatic cancer. Moreover, we detected ARL4C and BTG2 expression in pancreatic cancer tumor tissues and adjacent normal tissues and found that BTG2 was downregulated and ARL4C was upregulated in pancreatic cancer tumor tissues compared with adjacent normal tissues (Figures 1E,F). Therefore, circRNA_000864 was speculated to mediate BTG2 via miR-361-3p to influence pancreatic cancer development.

### CircRNA_000864 Is Poorly Expressed in Pancreatic Cancer Tissues and Cells

**FIGURE 2:**
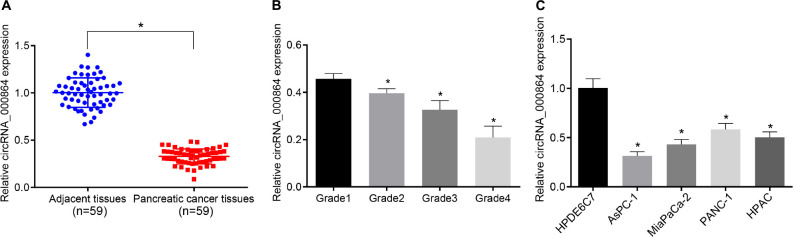
Downregulation of circRNA_000864 is observed in pancreatic cancer tissues and cells. **(A)** circRNA_000864 expression in pancreatic cancer tissues and adjacent normal tissues determined by RT-qPCR normalized to GAPDH (n = 59). **(B)** The expression of circRNA_000864 in different stages of pancreatic cancer (Grade 1 = 6, Grade 2 = 9, Grade 3 = 33, Grade 4 = 11). **(C)** The expression of circRNA_000864 in normal pancreatic epithelial cell lines and pancreatic cancer cell lines were detected by RT-qPCR normalized to GAPDH. *p < 0.05 *vs.* adjacent normal tissues or patients at Grade 1 or HPDE6C7 cells. The above data were all measurement data. The statistical results of **(A,B)** were expressed as mean ± standard deviation. Data of **(A)** were compared by the paired *t*-test, and data**of (B)** were compared by one-way ANOVA (Dunnett’s *post hoc* test). The statistical results in **(C)** were expressed as sample mean ± standard error and compared by using one-way ANOVA, and the cell experiment was conducted three times independently.

To investigate the expression changes of circRNA_000864 in pancreatic cancer, the circRNA_000864 expression pattern was detected in 59 pancreatic cancer tissues and adjacent normal tissues by RT-qPCR (Figure 2A). The results showed that the circRNA_000864 expression pattern in pancreatic cancer tissues was lower than the adjacent normal tissues (*p* < 0.05), and a higher degree of pancreatic cancer staging corresponded to a poor expression pattern of circRNA_000684 (*p* < 0.05; Figure 2B). Further experimental results also showed that compared with the normal pancreatic epithelial cell line HPDE6C7, circRNA_000864 expression was significantly decreased in the four pancreatic cancer cell lines AsPC-1, MiaPaCa-2, PANC-1, and HPAC, with the lowest expression pattern in AsPC-1 cells (*p* < 0.05; Figure 2C). Therefore, the AsPC-1 cells were selected for subsequent relevant experiments.

### Overexpression of CIrcRNA_000864 Inhibits the Proliferation, Invasion, and Cell Cycle, but Promotes Apoptosis of Pancreatic Cancer Cells and Inhibits Tumor Growth

**FIGURE 3:**
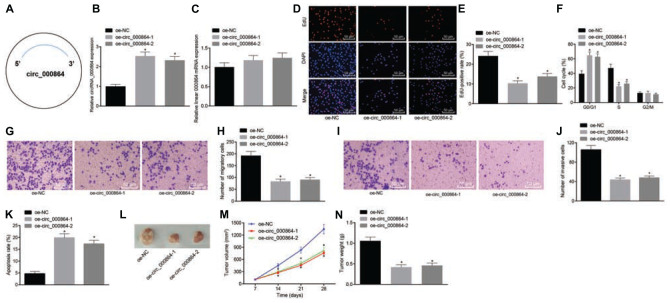
CircRNA_000864 upregulation results in suppression of pancreatic cell proliferation, migration, invasion, and antiapoptosis. AsPC-1 cells were transfected with oe-NC, oe-circRNA_00086-1, or oe-circRNA_00086-2. **(A)** A simple structure diagram of circRNA_000864. **(B)** CircRNA_000864 expression in AsPC-1 cells detected by RT-qPCR assay normalized to GAPDH. **(C)** Linear RNA_000864 expression in AsPC-1 cells by RT-qPCR normalized to GAPDH. **(D,E)** Proliferation of AsPC-1 cells detected by 5-ethynyl-2′-deoxyuridine (EdU) assay (×200). **(F)** Cell cycle distribution in AsPC-1 cells detected by flow cytometry. **(G,H)** Migration in AsPC-1 cells detected using Transwell assay (×200). **(I,J)** Invasion in AsPC-1 cells detected by Transwell assay (×200). **(K)** Apoptosis in AsPC-1 cells detected by flow cytometry. Mice were treated with oe-NC, oe-circRNA_00086-1, or oe-circRNA_00086-2. **(L)** Representative images of xenograft tumor formation in nude mice (*n* = 6). **(M)** Tumor volume of mice (*n* = 6). **(N)** Tumor weight of mice (*n* = 6). *p < 0.05 *vs.* AsPC-1 cells transfected with oe-NC. The above data were all measurement data. The statistical results of **(M,N)** were expressed as sample mean ± standard deviation. **(M)** was analyzed by repeated measurement ANOVA (Tukey’s *post hoc* test), and **(N)** was analyzed by ANOVA (Tukey’s *post hoc* test). The rest of the data were expressed as sample mean ± standard error. The cell experiment was conducted three times independently.

To assess whether circRNA_000864 affected the biological function of pancreatic cancer cells, the AsPC-1 cells were selected, which were then transfected with the oe-circRNA_000864 plasmid (Figure 3A). As demonstrated by RT-qPCR, circRNA_000864 expression was increased in AsPC-1 cells transfected with oe-circRNA_00086-1 or oe-circRNA_00086-2 (Figure 3B), but linear RNA_000864 expression had no significant differences in AsPC-1 cells transfected with oe-circRNA_00086-1 or oe-circRNA_00086-2 (Figure 3C). It indicated that the overexpression vector was successfully transfected into AsPC-1 cells.

Then, EdU assay, Transwell assay, and flow cytometry were conducted. The treatment of oe-circRNA_00086-1 or oe-circRNA_00086-2 reduced cell proliferation (Figures 3D,E), elevated cells arrested in the G0/G1 phase, but reduced the proportion of cells in the S phase (Figure 3F), declined cell migration and invasion (Figures 3G–J), and enhanced cell apoptosis (Figure 3K) in AsPC-1 cells. In addition, in order to figure out the effect of circRNA_000864 on pancreatic cancer *in vivo*, we injected the transfected AsPC-1 cells into nude mice and measured the tumor volume after injection. The experimental results (Figures 3L–N) presented that the tumor volume gradually increased with time. Meanwhile, the average tumor volume and weight were decreased after treatment of oe-circRNA_00086-1 or oe-circRNA_00086-2. The preceding results were reproduced in the study of MiaPaCa-2 cells (Supplementary Figure 1). The aforementioned results suggested that overexpression of circRNA_000864 repressed proliferation, migration, and invasion, but promoted the apoptosis of pancreatic cancer cells.

### CircRNA_000864 Binds to MiR-361-3p

**FIGURE 4:**
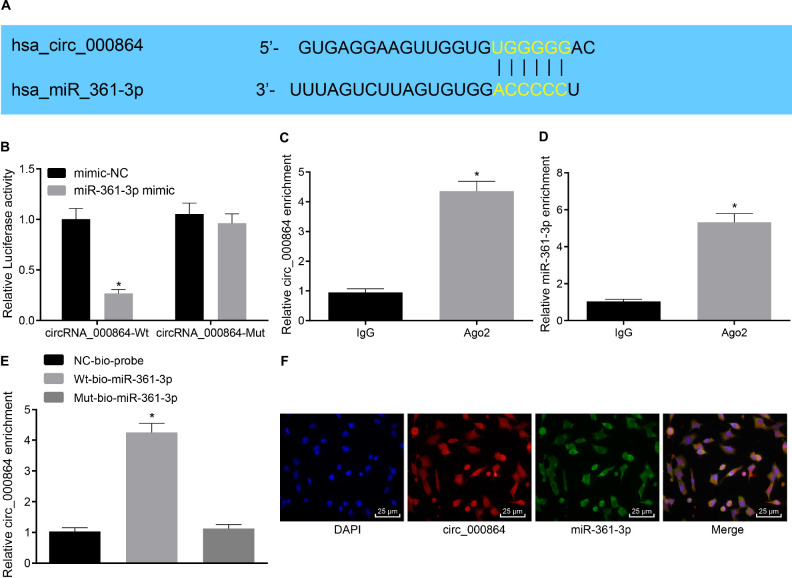
MiR-361-3p combines with circRNA_000864. **(A)** The binding site between circRNA_000864 and miR-361-3p predicted by biology websites. **(B)** The binding relationship between circRNA_000864 and miR-361-3p verified by dual-luciferase reporter gene assay. **(C)** The binding between circRNA_000864 and Argonaute 2 (Ago2) detected by RNA Immunoprecipitation (RIP) assay. **(D)** The binding between miR-361-3p and Ago2 determined by RIP assay. **(E)** The binding between circRNA_000864 and miR-361-3p measured by RNA-pull down assay. **(F)** Colocalization of circRNA_000864 with miR-361-3p in the cytoplasm evaluated by fluorescence *in situ* hybridization (FISH) assay (×400). *p < 0.05 vs. AsPC-1 cells transfected with mimic-NC or IgG or Mut-miR-361-3p. The above data were all measurement data, and the statistical results are expressed as sample mean ± standard deviation. **(E)** Data were compared by one-way ANOVA (Dunnett’s *post hoc* test), and data of the remaining figures were analyzed using the unpaired *t*-test. The experiment was conducted three times independently.

Subsequently, our aim shifted at investigating the downstream mechanism of circRNA_000864 in pancreatic cancer. A potential binding site between circRNA_000864 and miR-361-3p was predicted by the biological website CircInteractome (Figure 4A), and their targeting relationship was verified with dual-luciferase reporter gene assay. The results (Figure 4B) exhibited that the luciferase activity in the binding region of miR-361-3p and circRNA_000864-Wt was significantly inhibited (*p* < 0.05), while no significant change was evident in the luciferase activity of circRNA_000864-Mut (*p* > 0.05). Our results indicated that miR-361-3p could specifically bind to circRNA_000864. Meanwhile, co-immunoprecipitation assay (Figures 4C,D) showed that compared with IgG, circRNA_000864- and miR-361-3p-bound Ago2 was significantly increased (*p* < 0.05), which revealed that both the circRNA_000864 and miR-361-3p could bind with the Ago2 protein. Furthermore, the RNA-pull down assay (Figure 4E) demonstrated that compared with Mut-miR-361-3p, circRNA_000864 bound with Wt-miR-361-3p was elevated (*p* < 0.05), which indicated that miR-361-3p could directly integrate with circRNA_000864. Additionally, FISH analysis in the pancreatic cancer cells showed that circRNA_000864 and miR-361-3p were colocalized in the cytoplasm (Figure 4F). The aforementioned results showed that circRNA_000864 could successfully combine with miR-361-3p.

### CircRNA_000864 Represses Proliferation, Invasion, and Migration and Promotes Apoptosis of Pancreatic Cancer Cells by Binding to MiR-361-3p

**FIGURE 5:**
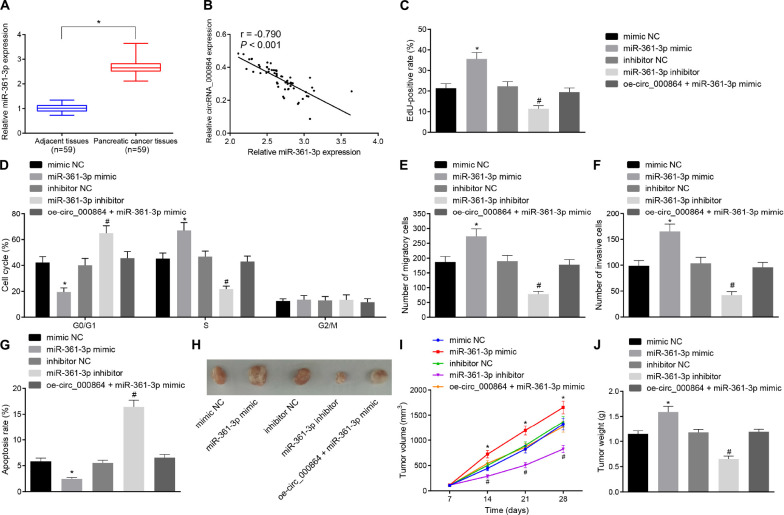
Downregulation of miR-361-3p contributes to inhibition of proliferation, migration, invasion, and antiapoptosis of pancreatic cancer cells. **(A)** RT-qPCR detection of relative expression levels of miR-361-3p in pancreatic cancer tissues and adjacent normal tissues normalized to U6 (*n* = 59). **(B)** Pearson correlation analysis of the correlation between circRNA_000864 and miR-361-3p (*n* = 59). AsPC-1 cells were transfected with mimic-NC, miR-361-3p mimic, inhibitor-NC, miR-361-3p inhibitor, or oe-circRNA_000864 + miR-361-3p mimic. **(C)** AsPC-1 cell proliferation detected by using EdU assay. **(D)** AsPC-1 cell cycle distribution detected by flow cytometry. **(E)** AsPC-1 cell migration detected by Transwell assay. **(F)** AsPC-1 cell invasion detected by Transwell assay. **(G)** AsPC-1 cell apoptosis detected by flow cytometry. Mice were treated with mimic-NC, miR-361-3pmimic, inhibitor-NC, miR-361-3p inhibitor, or oe-circRNA_000864 + miR-361-3pmimic. **(H)** Representative images of xenograft tumor in nude mice (*n* = 6). **(I)** Tumor volume of mice (*n* = 6). **(J)** Tumor weight of mice (*n* = 6). *p < 0.05 vs. adjacent normal tissues or AsPC-1 cells or mice treated with mimic-NC. ^#^*p* < 0.05 vs. AsPC-1 cells or mice treated with inhibitor-NC. The measurement data were expressed as sample mean ± standard deviation. Data of **(A)** were compared by the paired *t*-test, data of **(I)** were compared by repeated measures ANOVA (Tukey’s *post hoc* test), and data of **(J)** were compared by one-way ANOVA (Tukey’s *post hoc* test). The remaining statistical results were expressed as sample mean ± standard deviation and analyzed by using a one-way ANOVA (Tukey’s *post hoc* test). The cell experiment was conducted three times independently.

To investigate whether miR-361-3p affects the biological function of pancreatic cancer, the AsPC-1 cells were treated with the miR-361-3p mimic, miR-361-3p inhibitor, and oe-circRNA_000864. Initially, RT-qPCR was performed to determine the miR-361-3p expression pattern in the pancreatic cancer tissues, which revealed that miR-361-3p was highly expressed in the pancreatic cancer tissues (Figure 5A). Additionally, the results of correlation analysis showed a negative correlation between circRNA_000864 and miR-361-3p expression (Figure 5B).

Subsequently, EdU assay, Transwell assay, and flow cytometry were performed. As depicted in Figures 5C–G, the cell proliferation was elevated, the proportion of cells arrested in the G0/G1 phase decreased, while the proportion of cells in the S phase was increased, the cell migration and invasion were increased, and the cell apoptosis was reduced in AsPC-1 cells treated with miR-361-3p mimic, which was contradictory after treatment with miR-361-3p inhibitor. Furthermore, the oe-circRNA_000864 treatment abrogated the effect of miR-361-3p on AsPC-1 cell proliferation, cycle entry, apoptosis, migration, and invasion. The transfected AsPC-1 cells were then injected into the nude mice, followed by measurement of the tumor volume after administering the injection (Figures 5H–J). The findings revealed that the average tumor volume and weight were all significantly elevated after miR-361-3p mimic treatment, which was obliterated by circRNA_000864 overexpression. However, treatment with the miR-361-3p inhibitor resulted in the reduction of tumor volume and weight. The preceding results showed that circRNA_000864 could inhibit the cell proliferation, migration, invasion ability, cell cycle progression, and tumor growth of pancreatic cancer cells and promote cell apoptosis through binding to miR-361-3p.

### CircRNA_000864 Promotes BTG2 by Binding to MiR-361-3p

**FIGURE 6:**
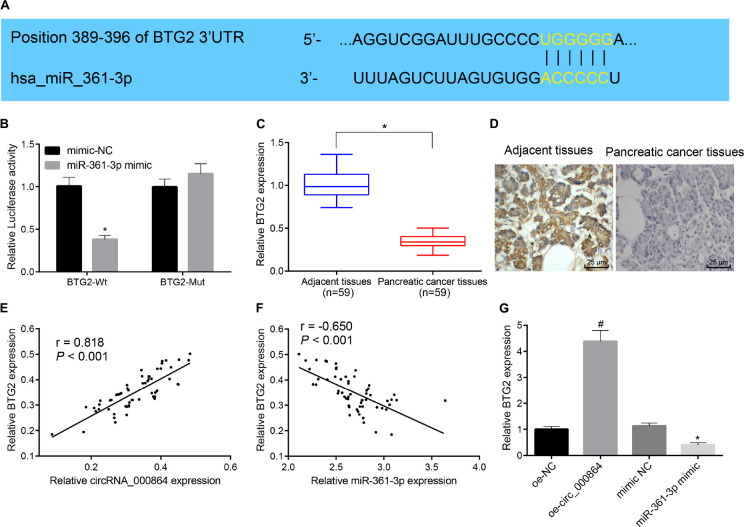
CircRNA_000864 binds to miR-361-3p to upregulate BTG2. **(A)** The predicted binding site of miR-361-3p and BTG2 through the biological website. **(B)** The targeting relationship between miR-361-3p and BTG2 assessed by dual-luciferase reporter gene assay. **(C)** BTG2 expression in pancreatic cancer tissues and adjacent normal tissues detected by RT-qPCR normalized to GAPDH (*n* = 59). **(D)** BTG2 expression in pancreatic cancer tissues and adjacent normal tissues detected by immunohistochemistry (×400) (*n* = 59). **(E)** The correlation analysis of circRNA_000864 and BTG2. **(F)** The correlation analysis of miR-361-3p and BTG2. **(G)** BTG2 expression in AsPC-1 cells after overexpression of circRNA_000864 and miR-361-3p detected by RT-qPCR normalized to GAPDH. * *p* < 0.05 vs. adjacent normal tissues or AsPC-1 cells transfected with mimic-NC. ^#^*p* < 0.05 *vs*. AsPC-1 cells transfected with oe-NC. The above data were all measurement data. The statistical results of **(B–G)** were expressed as sample mean ± standard deviation and analyzed by the unpaired *t*-test. The remaining results were expressed as mean ± standard deviation. Data of **(C)** were compared by the paired *t*-test. The cell experiment was conducted three times independently.

To explore the downstream regulatory target gene of miR-361-3p, we adopted TargetScan prediction and found that miR-361-3p had a binding site with BTG2 in the pancreatic cancer cells (Figure 6A). Then, the results of the dual-luciferase reporter gene assay (Figure 6B) showed that the luciferase activity of the BTG2-3′UTR-Wt could be inhibited by miR-361-3p mimic (*p* < 0.05), while the luciferase activity of the BTG2-3′UTR-MUT remained unchanged (*p* > 0.05). This indicated that miR-361-3p could specifically bind with the 3′UTR region of BTG2 and thus downregulate BTG2. Then, RT-qPCR and immunohistochemistry were used to detect BTG2 expression in pancreatic cancer tissues and adjacent normal tissues. The results (Figures 6C,D) exhibited that BTG2 was lowly expressed in pancreatic cancer tissues. Moreover, correlation analysis indicated that circRNA_000864 was positively correlated with BTG2 (Figure 6E), while miR-361-3p was negatively correlated with BTG2 (Figure 6F). Then, the results of RT-qPCR exhibited that the BTG2 mRNA level was elevated by overexpressing circRNA_000864 but reduced by miR-361-3p mimic treatment, while no significant change was evident between oe-circRNA_000864 and miR-361-3p mimic treatment (Figure 6G). The aforementioned results elicited BTG2 as a direct target gene of miR-361-3p, where the overexpression of circRNA_000864 can indirectly induce the overexpression of BTG2 by binding to miR-361-3p.

### CircRNA_000864-Upregulated BTG2 Suppresses Proliferation, Invasion, and Migration and Induces Apoptosis of Pancreatic Cancer Cells

**FIGURE 7:**
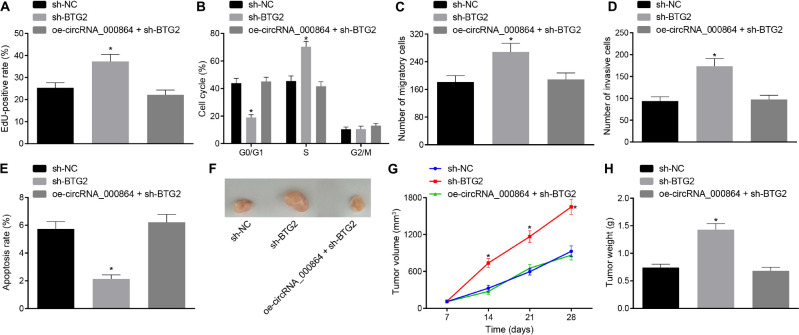
CircRNA_000864 represses proliferation, migration, invasion, and antiapoptosis of pancreatic cancer cells by upregulating BTG2. AsPC-1 cells were transfected with sh-NC, sh-BTG2, or oe-circRNA_000864 + sh-BTG2. **(A)** AsPC-1 cell proliferation detected by EdU assay. **(B)** AsPC-1 cell cycle distribution detected by flow cytometry. **(C)** AsPC-1 cell migration detected by Transwell assay. **(D)** AsPC-1 cell invasion detected by Transwell assay. **(E)** AsPC-1 cell apoptosis detected by flow cytometry. Mice were treated with sh-NC, sh-BTG2, or oe-circRNA_000864 + sh-BTG2. **(F)** Representative images of xenograft tumor formation in nude mice (*n* = 6). **(G)** Tumor volume of mice (*n* = 6). **(H)** Tumor weight of mice (*n* = 6). *p < 0.05 vs. AsPC-1 cells transfected with sh-NC. The above data were all measurement data. Data of **(G,H)** were expressed as mean ± standard deviation. Data of **(G)** were analyzed by repeated measures ANOVA (Tukey’s *post hoc* test), and data of **(H**) were compared using one-way ANOVA (Tukey’s *post hoc* test). The remaining statistical results were summarized as sample mean ± standard error. The rest of the data were compared by using unpaired *t*-test. The cell experiment was conducted three times independently.

In order to study the effect of circRNA_000864 regulating BTG2 on the biological function of the pancreatic cancer cells, we initially interfered with the BTG2 expression (Supplementary Figure 2) and then tested the proliferation, migration and invasion ability, cell cycle progression, and apoptosis. The treatment with sh-BTG2 resulted in enhanced cell proliferation (Figure 7A), reduced cells arrested in the G0/G1 phase (Figure 7B), increased migration and invasion (Figures 7C,D), and lowered cell apoptosis (Figure 7E) in the AsPC-1 cells, which was neutralized by oe-circRNA_000864 treatment. Xenotransplantation of nude mice documented that over an extended period of time, the volume of tumor increased gradually. At the same time, the average tumor volume and weight were significantly increased by silencing BTG2, which was abrogated by overexpressing circRNA_000864 (Figures 7F–H). The above results revealed that overexpression of circRNA_000864 can upregulate BTG2, thus inhibiting proliferation, migration, invasion ability, and cell cycle, and promoting apoptosis of pancreatic cancer cells, tumor formation, and growth *in vivo*.

## Discussion

Pancreatic cancer, a type of malignant digestive disease, elicits rapid growth and poor early prognosis, where cytotoxic agents remain the most widely adopted treatment protocol for pancreatic cancer irrespective of palliative or adjuvant treatment (17). Research has predicted the involvement of circular RNAs (circRNAs) in cancer progression and furthermore as diagnostic biomarkers of various diseases and providing novel treatments (18). Additionally, in this dissertation, circRNA_000864 overexpression impaired miR-361-3p-dependent BTG2 downregulation and inhibited the proliferation, migration, invasion, and cycle entry, and facilitated the apoptosis of pancreatic cells.

Our experimental results revealed a poor expression pattern of circRNA_000864 in pancreatic cancer tissues, and that overexpression of circRNA_000864 induced the cell cycle arrest of more pancreatic cancer cells in the G0/G1 phase, inhibited proliferation, migration, and invasion of pancreatic cancer cells, and stimulated cell apoptosis. Existing research documented that hsa_circRNA_100533 was downregulated in oral squamous cell carcinoma tissues, and the upregulated expression pattern of hsa_circRNA_100533 could suppress the cell proliferation, migration, and invasion, and uphold the cell apoptosis in oral squamous cell carcinoma (19). In colorectal cancer, hsa_circRNA_103809 (hsa_circ_0072088, circZFR) was poorly expressed, and hsa_circRNA_103809 contributes to the inhibition of the biological functions of colorectal cancer cells (20). However, circRNAs have dual functionality in pancreatic-related anomalies. For instance, has_circRNA_100782 can also be upregulated in PDAC (21), and an elevated expression pattern of circRHOT1 has been identified in pancreatic cancer (22). The underlying reason for the aberrant expression of circRNAs in pancreatic cancer asserts extensive investigation. In addition, research also demonstrated that upregulation of circ-0001649 inhibits the cell proliferation and migration of hepatocellular carcinoma (HCC) (23). Moreover, another study elucidated a poor expression pattern of hsa_circRNA_0001649 in PDAC tissues and cells, and that forced expression of hsa_circRNA_0001649 resulted in suppression of cell proliferation and colony formation and advancement of cell apoptosis in PADC (24). The aforementioned literature implicitly supported our results signifying the downregulation and the suppressive role of circRNA_000864 in pancreatic cancer development.

Another vital finding revealed that circRNA_000864 and miR-361-3p could directly bind with each other. Furthermore, some similar circRNAs also principally functioned as tumor suppressors by binding with certain miRs, which could promote cancer progression. Moreover, hsa_circ_103809 inhibits the cell proliferation, migration, and invasion in HCC by explicitly repressing the expression pattern of its target miR-620 (25). Our findings also ascertained a high miR-361-3p expression pattern in pancreatic cancer tissues. Additionally, the downregulation of miR-361-3p suppresses the proliferation, migration, and invasion, and cell cycle entry of pancreatic cancer cells and stimulates pancreatic cancer cell apoptosis. In advanced prostate cancer, miR-361-3p inhibitor demonstrated its functionality by reducing prostate cancer stem cell proliferation and increasing cell apoptosis (26). Downregulation of miR-299-3p suppresses pancreatic cancer malignant progression through the blockade of the Notch1 pathway (27). Consistently, miR-361-3p upregulation was observed in PDAC cells, and the ectopic expression of miR-361-3p stimulated the migration and invasion of PADC cells (11).

It is acknowledged that miRNAs can suppress the target mRNAs by binding to mRNAs at specific sites (28). For instance, miR-361-3p targeted DUSP2 to promote the development of PDAC (11). Furthermore, miR-6875-3p has been demonstrated to target BTG2 by specifically binding to 3′-UTR of BTG2 in HCC (29). The aforementioned findings implicitly support our findings validating BTG2 as a direct gene target of miR-361-3p. Moreover, in the current study, BTG2 decreased the proliferation, migration and invasion, cell cycle entry, and induced the apoptosis of pancreatic cancer cells. Meanwhile, a lower expression of BTG2 was evident in bladder cancer tissues compared to the normal bladder tissues (30). The overexpression of BTG2 significantly reduced the cell proliferation and tumorigenicity in HCC (31). Additionally, BTG2 was identified as a direct target gene of miR-27a-3p in gastric cancer, and overexpression of BTG2 arrested the cell cycle of gastric cancer cells at the G1 phase and induced cell apoptosis (32). BTG2 depression induces HCC cell proliferation (33).

## Conclusion

**FIGURE 8:**
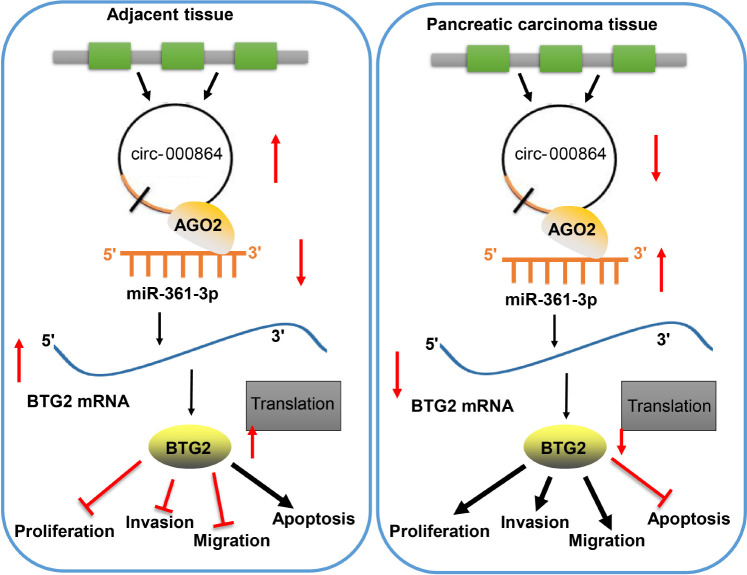
The mechanism of circRNA_000864 in pancreatic cancer by binding to miR-361-3p and BTG2. Upregulating circRNA_000864 expression could impair miR-361-3p-dependent BTG2 downregulation and inhibits the proliferation, invasion, migration, and promotes apoptosis of pancreatic cancer cells.

To conclude, circRNA_000864 and BTG2 exercise their antioncogenic effects on pancreatic cancer, while miR-361-3p exercises its oncogenic effects on pancreatic cancer. Upregulating circRNA_000864 expression could upregulate impaired BTG2 and inhibit the proliferation, invasion, migration, and antiapoptosis of pancreatic cancer cells by binding to miR-361-3p (Figure 8). Our findings indicated that circRNA_000864, miR-361-3p, and BTG2 could function as potential targets for the treatment of pancreatic cancer. However, adequately powered clinical trials testing the effect of circRNA_000864/miR-361-3p/BTG2 axis on pancreatic cancer treatment are lacking. Further, whether there are other downstream substrates of circRNA_000864 is still to be investigated. Therefore, additional studies are needed to validate our finding.

## Data Availability Statement

Publicly available datasets were analyzed in this study, these can be found in the NCBI Gene Expression Omnibus (GSE79634 and GSE16515).

## Ethics Statement

The animal study was reviewed and approved by the Experimental Animal Ethics Committee of Hubei University of Medicine. The studies involving human participants were reviewed and approved by the Ethics Committee of Hubei University of Medicine. The patients/participants provided their written informed consent to participate in this study.

## Author Contributions

LH and HY designed the study. LH, JH, HY, and JL collected the data. LH, LG, ZW, and XZ analyzed the data. LH, JH, HY, JL, SS, and GF interpreted the data. All authors drafted and revised the manuscript content, and approved the final version of the manuscript.

## Conflict of Interest

The authors declare that the research was conducted in the absence of any commercial or financial relationships that could be construed as a potential conflict of interest.

## Footnotes

1 http://www.circbase.org

2 https://www.ncbi.nlm.nih.gov/geo/

3 https://cran.r-project.org/web/packages/pheatmap/index.html

4 https://circinteractome.nia.nih.gov/index.html

5 http://www.targetscan.org/vert_71/

6 https://cm.jefferson.edu/rna22/

7 http://mirwalk.umm.uni-heidelberg.de/

8 http://www.exiqon.com/microrna-target-prediction

9 http://starbase.sysu.edu.cn/index.php

10 http://hoctar.tigem.it/

11 https://cran.r-project.org/web/packages/UpSetR/index.html

12 http://www.elisakits.cn

13 https://web.expasy.org/cgi-bin/cellosaurus/search

## Supplementary Material

Supplementary Figure 1 CircRNA_000864 upregulation results in suppression of pancreatic cell proliferation, migration, invasion and anti-apoptosis. MiaPaCa-2 cells were transfected with oe-NC, oe-circRNA_00086-1 or oe-circRNA_00086-2. A, CircRNA_000864 expression and Linear RNA_000864 expression in AsPC-1 cells detected by RT-qPCR assay normalized to GAPDH. B, Proliferation of AsPC-1 cells detected by EdU assay (×200). C, Cell cycle distribution in AsPC-1 cells detected by flow cytometry. D, Migration in AsPC-1 cells detected using Transwell assay (×200). E, Invasion in AsPC-1 cells detected by Transwell assay (×200). F, Apoptosis in AsPC-1 cells detected by flow cytometry. Mice were treated with oe-NC, oe-circRNA_00086-1 or oe-circRNA_00086-2. G, Representative images of xenograft tumor formation in nude mice, tumor volume and weight of mice (*n* = 6). **p* < 0.05 *vs.* AsPC-1 cells transfected with oe-NC. The above data were all measurement data. The statistical results of Figure G were expressed as sample mean ± standard deviation. Tumor growth curve was analyzed by repeated measurement ANOVA (Tukey’s *post hoc* test). Tumor weight was analyzed by one-way ANOVA (Tukey’s *post hoc* test). The rest data were expressed as sample mean ± standard error. The experiment was conducted three times independently.

Supplementary Figure 2 BTG2 silence efficiency. **p* < 0.05 *vs*. cells transfected with sh-NC.

## References

[1] Bray F Ferlay J Soerjomataram I Siegel RL Torre LA Jemal A. Global cancer statistics 2018: GLOBOCAN estimates of incidence and mortality worldwide for 36 cancers in 185 countries. *CA Cancer J Clin*. (2018) 68:394–424. 10.3322/caac.21492 30207593

[2] Wood LD Yurgelun MB Goggins MG. Genetics of familial and sporadic pancreatic cancer. *Gastroenterology*. (2019) 156:2041–55. 10.1053/j.gastro.2018.12.039 30660730

[3] Ogrendik M. Periodontal pathogens in the etiology of pancreatic cancer. *Gastrointest Tumors*. (2017) 3:125–7. 10.1159/000452708 28611978 PMC5465713

[4] Neoptolemos JP Kleeff J Michl P Costello E Greenhalf W Palmer DH. Therapeutic developments in pancreatic cancer: current and future perspectives. *Nat Rev Gastroenterol Hepatol*. (2018) 15:333–48. 10.1038/s41575-018-0005-x 29717230

[5] Vennin C Murphy KJ Morton JP Cox TR Pajic M Timpson P. Reshaping the tumor stroma for treatment of pancreatic cancer. *Gastroenterology*. (2018) 154:820–38. 10.1053/j.gastro.2017.11.280 29287624

[6] Chen Y Li C Tan C Liu X. Circular RNAs: a new frontier in the study of human diseases. *J Med Genet*. (2016) 53:359–65. 10.1136/jmedgenet-2016-103758 26945092

[7] Xu Y Yao Y Gao P Cui Y. Upregulated circular RNA circ_0030235 predicts unfavorable prognosis in pancreatic ductal adenocarcinoma and facilitates cell progression by sponging miR-1253 and miR-1294. *Biochem Biophys Res Commun*. (2019) 509:138–42. 10.1016/j.bbrc.2018.12.088 30591218

[8] Yang F Liu DY Guo JT Ge N Zhu P Liu X Circular RNA circ-LDLRAD3 as a biomarker in diagnosis of pancreatic cancer. *World J Gastroenterol*. (2017) 23:8345–54. 10.3748/wjg.v23.i47.8345 29307994 PMC5743505

[9] Guo S Xu X Ouyang Y Wang Y Yang J Yin L Microarray expression profile analysis of circular RNAs in pancreatic cancer. *Mol Med Rep*. (2018) 17:7661–71. 10.3892/mmr.2018.8827 29620241 PMC5983963

[10] Chen L Nan A Zhang N Jia Y Li X Ling Y Circular RNA 100146 functions as an oncogene through direct binding to miR-361-3p and miR-615-5p in non-small cell lung cancer. *Mol Cancer*. (2019) 18:13. 10.1186/s12943-019-0943-0 30665425 PMC6340182

[11] Hu J Li L Chen H Zhang G Liu H Kong R MiR-361-3p regulates ERK1/2-induced EMT via DUSP2 mRNA degradation in pancreatic ductal adenocarcinoma. *Cell Death Dis*. (2018) 9:807. 10.1038/s41419-018-0839-8 30042387 PMC6057920

[12] Du L Borkowski R Zhao Z Ma X Yu X Xie XJ A high-throughput screen identifies miRNA inhibitors regulating lung cancer cell survival and response to paclitaxel. *RNA Biol*. (2013) 10:1700–13. 10.4161/rna.26541 24157646 PMC3907480

[13] Yuniati L Scheijen B van der Meer LT van Leeuwen FN. Tumor suppressors BTG1 and BTG2: Beyond growth control. *J Cell Physiol*. (2019) 234:5379–89. 10.1002/jcp.27407 30350856 PMC6587536

[14] Chen H Pan H Qian Y Zhou W Liu X. MiR-25-3p promotes the proliferation of triple negative breast cancer by targeting BTG2. *Mol Cancer*. (2018) 17:4. 10.1186/s12943-017-0754-0 29310680 PMC5759260

[15] Arocho A Chen B Ladanyi M Pan Q. Validation of the 2-DeltaDeltaCt calculation as an alternate method of data analysis for quantitative PCR of BCR-ABL P210 transcripts. *Diagn Mol Pathol*. (2006) 15:56–61. 10.1097/00019606-200603000-00009 16531770

[16] Feng X Liu C Zhong D Xu D Ning C Wang J. [Influence of Immunohistochemistry scoring criteria in detecting EGFR Mutations]. *Zhongguo Fei Ai Za Zhi*. (2015) 18:740–4. 10.3779/j.issn.1009-3419.2015.12.05 26706950 PMC6015181

[17] Shi S Yu X. Selecting chemotherapy for pancreatic cancer: Far away or so close? *Semin Oncol*. (2019) 46:39–47. 10.1053/j.seminoncol.2018.12.004 30611527

[18] Yin Y Long J He Q Li Y Liao Y He P Emerging roles of circRNA in formation and progression of cancer. *J Cancer*. (2019) 10:5015–21. 10.7150/jca.30828 31602252 PMC6775606

[19] Zhu X Shao P Tang Y Shu M Hu WW Zhang Y. hsa_circRNA_100533 regulates GNAS by sponging hsa_miR_933 to prevent oral squamous cell carcinoma. *J Cell Biochem*. (2019) 120:19159–71. 10.1002/jcb.29245 31297884

[20] Bian L Zhi X Ma L Zhang J Chen P Sun S Hsa_circRNA_103809 regulated the cell proliferation and migration in colorectal cancer via miR-532-3p / FOXO4 axis. *Biochem Biophys Res Commun*. (2018) 505:346–52. 10.1016/j.bbrc.2018.09.073 30249393

[21] Chen G Shi Y Zhang Y Sun J. CircRNA_100782 regulates pancreatic carcinoma proliferation through the IL6-STAT3 pathway. *Onco Targets Ther*. (2017) 10:5783–94. 10.2147/OTT.S150678 29255366 PMC5722018

[22] Qu S Hao X Song W Niu K Yang X Zhang X Circular RNA circRHOT1 is upregulated and promotes cell proliferation and invasion in pancreatic cancer. *Epigenomics*. (2019) 11:53–63. 10.2217/epi-2018-0051 30444423

[23] Su Y Xu C Liu Y Hu Y Wu H. Circular RNA hsa_circ_0001649 inhibits hepatocellular carcinoma progression via multiple miRNAs sponge. *Aging (Albany N Y)*. (2019) 11:3362–75. 10.18632/aging.101988 31137016 PMC6813922

[24] Jiang Y Wang T Yan L Qu L. A novel prognostic biomarker for pancreatic ductal adenocarcinoma: hsa_circ_0001649. *Gene*. (2018) 675:88–93. 10.1016/j.gene.2018.06.099 29969694

[25] Li X Shen M. Circular RNA hsa_circ_103809 suppresses hepatocellular carcinoma proliferation and invasion by sponging miR-620. *Eur Rev Med Pharmacol Sci*. (2019) 23:555–66. 10.26355/eurrev_201902_16868 30720163

[26] Fletcher CE Sulpice E Combe S Shibakawa A Leach DA Hamilton MP Androgen receptor-modulatory microRNAs provide insight into therapy resistance and therapeutic targets in advanced prostate cancer. *Oncogene*. (2019) 38:5700–24. 10.1038/s41388-019-0823-5 31043708 PMC6755970

[27] Xu K Zhang L. Inhibition of TUG1/miRNA-299-3p axis represses pancreatic cancer malignant progression via suppression of the notch1 pathway. *Dig Dis Sci*. (2019) 65:1748–60. 10.1007/s10620-019-05911-0 31655908

[28] John B Enright AJ Aravin A Tuschl T Sander C Marks DS. Human MicroRNA targets. *PLoS Biol*. (2004) 2:e363. 10.1371/journal.pbio.0020363 15502875 PMC521178

[29] Xie Y Du J Liu Z Zhang D Yao X Yang Y. MiR-6875-3p promotes the proliferation, invasion and metastasis of hepatocellular carcinoma via BTG2/FAK/Akt pathway. *J Exp Clin Cancer Res*. (2019) 38:7. 10.1186/s13046-018-1020-z 30621734 PMC6323674

[30] Tsui KH Chiang KC Lin YH Chang KS Feng TH Juang HH. BTG2 is a tumor suppressor gene upregulated by p53 and PTEN in human bladder carcinoma cells. *Cancer Med*. (2018) 7:184–95. 10.1002/cam4.1263 29239139 PMC5773943

[31] Huang CS Zhai JM Zhu XX Cai JP Chen W Li JH BTG2 is down-regulated and inhibits cancer stem cell-like features of side population cells in hepatocellular carcinoma. *Dig Dis Sci*. (2017) 62:3501–10. 10.1007/s10620-017-4829-y 29098552

[32] Zhou L Liang X Zhang L Yang L Nagao N Wu H MiR-27a-3p functions as an oncogene in gastric cancer by targeting BTG2. *Oncotarget*. (2016) 7:51943–54. 10.18632/oncotarget.10460 27409164 PMC5239526

[33] Jiang H Zhu Y Zhou Z Xu J Jin S Xu K PRMT5 promotes cell proliferation by inhibiting BTG2 expression via the ERK signaling pathway in hepatocellular carcinoma. *Cancer Med*. (2018) 7:869–82. 10.1002/cam4.1360 29441724 PMC5852340

