# Supplementary figures and images for: CircRNA_000864 Upregulates B-cell Translocation Gene 2 Expression and Represses Migration and Invasion in Pancreatic Cancer Cells by Binding to miR-361-3p
Source: Front Oncol. 2020 Dec 23;10:547942. doi: 10.3389/fonc.2020.547942 (PMC7793745; doi:10.3389/fonc.2020.547942)

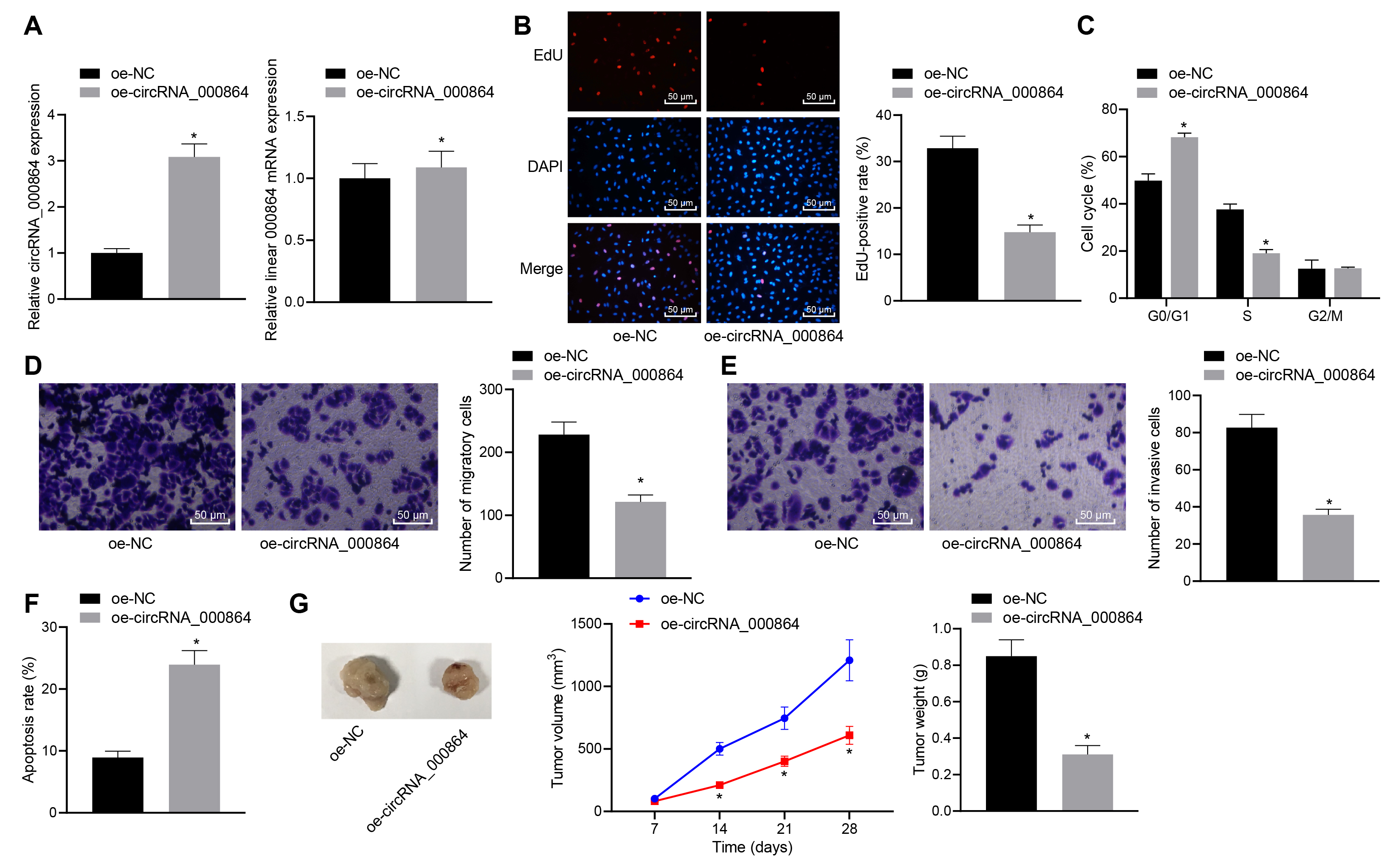

Supplement: Supplementary Figure 1 — CircRNA_000864 upregulation results in suppression of pancreatic cell proliferation, migration, invasion and anti-apoptosis. MiaPaCa-2 cells were transfected with oe-NC, oe-circRNA_00086-1 or oe-circRNA_00086-2. A, CircRNA_000864 expression and Linear RNA_000864 expression in AsPC-1 cells detected by RT-qPCR assay normalized to GAPDH. B, Proliferation of AsPC-1 cells detected by EdU assay (×200). C, Cell cycle distribution in AsPC-1 cells detected by flow cytometry. D, Migration in AsPC-1 cells detected using Transwell assay (×200). E, Invasion in AsPC-1 cells detected by Transwell assay (×200). F, Apoptosis in AsPC-1 cells detected by flow cytometry. Mice were treated with oe-NC, oe-circRNA_00086-1 or oe-circRNA_00086-2. G, Representative images of xenograft tumor formation in nude mice, tumor volume and weight of mice (n = 6). *p < 0.05 vs. AsPC-1 cells transfected with oe-NC. The above data were all measurement data. The statistical results of Figure G were expressed as sample mean ± standard deviation. Tumor growth curve was analyzed by repeated measurement ANOVA (Tukey’s post hoc test). Tumor weight was analyzed by one-way ANOVA (Tukey’s post hoc test). The rest data were expressed as sample mean ± standard error. The experiment was conducted three times independently. [file Image_1.JPEG]

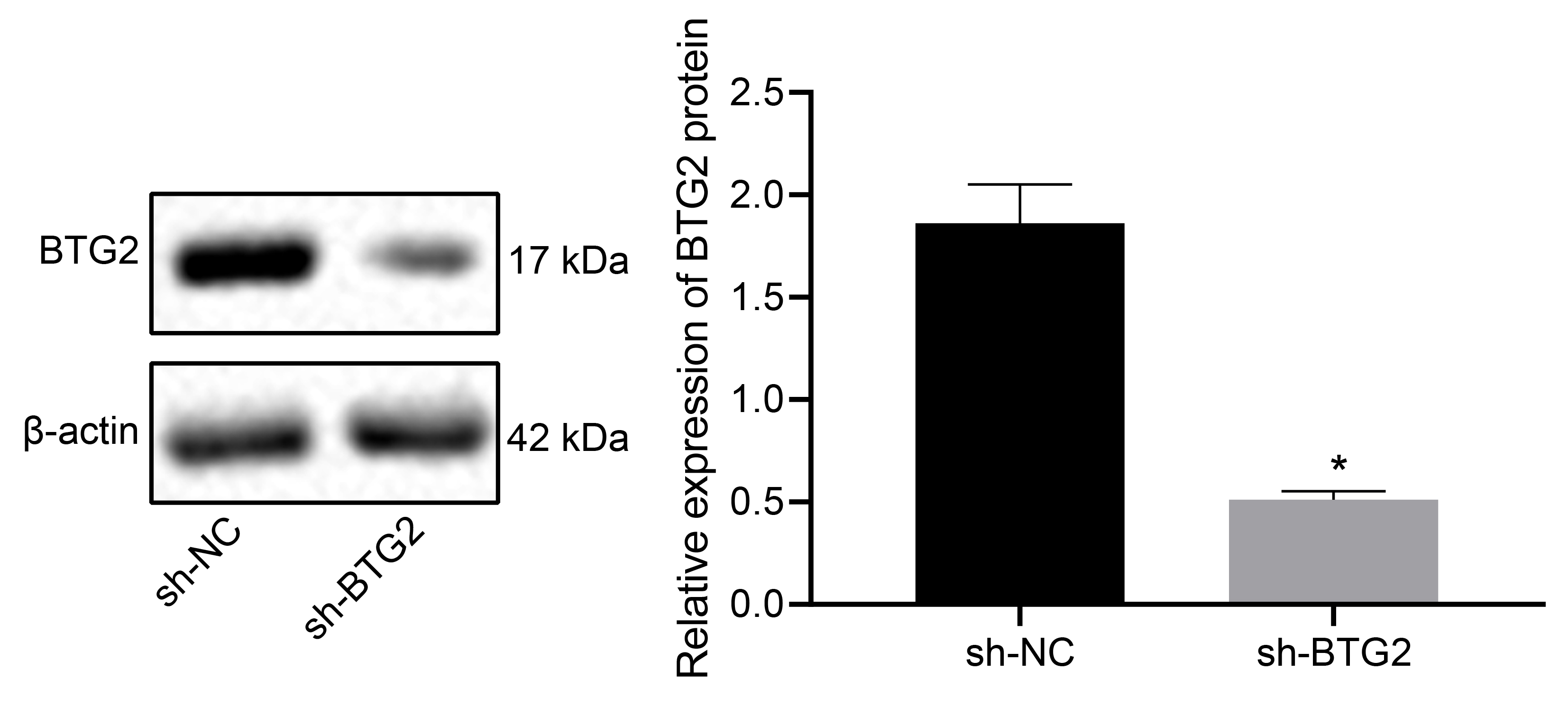

Supplement: Supplementary Figure 2 — BTG2 silence efficiency. *p < 0.05 vs. cells transfected with sh-NC. [file Image_2.JPEG]
